# Location attributes explaining the entry of firms in creative industries: evidence from France

**DOI:** 10.1007/s00168-022-01196-w

**Published:** 2023-04-05

**Authors:** Josep-Maria Arauzo-Carod, Eva Coll-Martínez, Camelia Turcu

**Affiliations:** 1 Laboratoire d’Économie d’Orléans, Université d’Orléans, Rue de Blois, BP 26739, 45067 Orléans, France; 2 Sciences Po Toulouse, Manufacture Des Tabacs, LEREPS, Université de Toulouse, 21 Allée de Brienne - CS 88526, 31685 Toulouse cedex 6, France; 3 ECO-SOS & QURE, Departament d’Economia, Universitat Rovira I Virgili, Av. Universitat, 1, 43204 Reus, Catalonia, Spain

**Keywords:** R39, Z100

## Abstract

This paper focuses on creative industries and the role played by the existing spatial distribution and agglomeration economies of these activities in relation to their entry decisions. We rely on employment and firm-level data in the creative industries (provided by INSEE) and compare the location of new establishments in the creative and non-creative industries between 2009 and 2013 in French departments (NUTS 3 regions). We use count data models and spatial econometrics to show that location determinants are rather similar in creative and non-creative industries and that specialisation in creative industries positively influences the entry of all other industries. The French case provides new insights to understand the geographical patterns of creative industries.

## Introduction

Considerable attention has been devoted in the economic literature to the factors that influence the location decisions of new firms (Arauzo-Carod et al. 2010). Existing work attempts to identify and quantify the determinants of entry and tends to focus either on specified industries aggregated over regions or, conversely, on aggregated industry sectors (manufacturing, services) in specific geographical regions. More detailed analyses, with both industries and regions being specified, would be of great interest in helping to elucidate spatial and industry-specific characteristics. These, however, are much scarcer.

In addition, in developed countries there are some activities that have noticeably seen an increase in weight in overall economic activity very recently. Unfortunately, however, they have not received enough attention from the academic community in order to understand forces driving entry of firms and, especially, their location decisions when choosing among alternative territories. This paper, therefore, focuses on Creative Industries (CIs), a group of industries linked to cultural, creative, and high-tech activities^1^ that have experienced high growth rates in recent years and that have relevant positive externalities (Sanchez-Serra 2014). They contribute to knowledge generation and the prestige of areas specialised in these activities (Myerscough, 1988). This, in turn, may attract other firms and economic activity (Gutierrez-Posada et al. 2021; Bille and Schulze 2006), boost regional employment growth (Piergiovanni et al. 2012) and the productivity of existent firms (Coll-Martinez and Arauzo-Carod 2019). Key works highlighting the positive perception of CIs include two contributions by Florida (2005, 2002), who he has provided a measure of a “creative class” and a first (qualitative) attempt to quantify its contribution over economic activity.

The current understanding of CI entry determinants is quite limited and further work is necessary on the processes that drive their entry. This paper aims to partially address this gap by analysing the French case at the province (*département,* or NUTS 3) level. This is of special interest in view of the importance of CIs in terms of (i) the number of firms and employees (IFM 2013), (ii) the growth of workforce in CIs (Chantelot 2010a), (iii) the strong export profile of firms, and (iv) the fact that (despite some concentration in the Paris region), there is a relatively well-balanced territorial distribution—despite being noticeably heterogeneous, all departments generate and attract new CIs. In addition, French CIs have a worldwide reputation since they include some globally prominent actors in areas that include fashion design, arts and entertainment, and publishing (Scott 2000; APUR 2014). There are also important inter-industry linkages arising from CIs as they contribute to the prestige of certain areas and attract firms from quite different and unrelated industries (Coll-Martínez and Arauzo-Carod 2017). Understanding what determines CI location choices is crucial in designing public policies aiming at attracting innovative firms to French regions.

Our econometric results, obtained using Panel Count Data Models for French departments, suggest that on average, the probability of a creative firm locating in a French department increases with the amount of human capital, disposable income per inhabitant, the unemployment rate, a number of cultural amenities such as museums and cinemas. This probability diminishes with the share of manufacturing activities, public investment per inhabitant, distance to Paris, and weather factors (proxied by cumulative rainfall). We found that both creative and non-creative firms are positively influenced by the specialisation level of creative industries. However, when considering neighbouring effects, the impact of CIs does not extend beyond the borders of the department.

The paper is organised as follows. In the second section, we discuss theoretical and empirical contributions regarding firm entry and CIs, and we focus on those that specifically analyse entries in these industries and that consider spatial factors. In the third section, we present the methodology and the econometric specification. In the fourth section, we describe the data and variables. In the fifth section, we discuss the main results. We present our conclusions in the sixth and final section.

## Literature review: firm entry and CIs

Understanding firm entry decisions is becoming more and more relevant for policy makers as new firms are commonly hypothesised to be drivers of a wide range of positive effects that include local and regional development (Acs et al. 2009), regional diversity (Noseleit 2015), technological change (Rigby and Essletzbichler 2000), productivity growth (Brixy 2014) and innovation (Audretsch 1995). Initially, the analysis of these decisions focused solely on the industry-specific determinants without including a spatial dimension (Orr 1974). Spatial asymmetries, however, make some territories significantly more attractive than others and, since the mid-nineties, the spatial dimension has received more attention (Reynolds et al. 1994).

Accordingly, empirical contributions focusing on aggregate firm entries (typically restricted to manufacturing industries but, to some extent, also to services) have identified several spatially specific entry determinants. The most well-known of these are agglomeration economies (Fotopoulos and Louri 2000), entrepreneurial attitude (Bosma and Schutjens 2011), firm structure (Arauzo-Carod and Segarra-Blasco 2005; Kangasharju 2000), population size (Armington and Acs 2002), institutional quality (Acs et al. 2008), income (Elert 2014), human capital (Armington and Acs 2002), persistence of previous entries (Andersson and Koster 2010), and labour market characteristics (Santarelli et al. 2009).

In the literature, “traditional” manufacturing or service activities have attracted much more attention than have CIs. When the latter were analysed, that attention has often been solely put on their role as magnets for other activities (Hall 2000), as promoters of firm entries (De Jong et al. 2007), or as tools for economic growth (De Propris 2013; Piergiovanni et al. 2012), rather than on the specific entry determinants for these industries. Nevertheless, empirical contributions on the location determinants for CIs do exist. These include the works of Coll-Martínez and Arauzo-Carod (2017) for Catalan municipalities, Coll-Martínez et al. (2019) for Barcelona at the intra-urban level, Kiroff (2017) for the design subsector in Auckland, Sanchez-Serra (2016) for Spanish travel-to-work areas, Boix et al. (2015) for a selection of European metropolitan areas, Wenting (2011) for fashion design firms in the Netherlands, Smit (2011) for three Dutch cities, Bertacchini and Borrione (2013) for Italian regions and Cruz and Teixeira (2014) for Portuguese municipalities.

Although the methodologies, geographical areas and the research focus of these studies differ considerably, some common key location determinants have been identified. Specifically, as distinct from traditional agglomeration economies (Sanchez-Serra 2016), specialisation in CIs is a strong determinant for entries of both creative and non-creative firms (Coll-Martínez and Arauzo-Carod, 2017). Similarly, there is empirical evidence indicating that all types of firms benefit from the existence of an intangible *creative milieu* favouring entries (Coll-Martínez and Arauzo-Carod 2017; Wojan et al. 2007) as well as creative externalities (Sanchez-Serra 2016). Previous results highlight the strong interindustry linkages between creative and non-creative industries that enhance the positive effects of the former over the latter. In this sense, recent contributions highlight that only the cross-fertilisation of different creative talents working in different fields may stimulate creativity, ultimately enhancing regional development (Bakhshi and McVittie 2009; Cerisola 2018a, b; Innocenti and Lazzeretti 2021). Empirical evidence also indicates a strong preference for CI co-located clusters where there are also non-creative activities (Boix et al. 2015). In terms of the locational preferences of CIs, they tend to agglomerate in metropolitan areas (Boix et al. 2015; Sanchez-Serra 2013, 2014) and, within that, try to benefit from agglomeration economies by concentrating close to core neighbourhoods (Coll-Martínez et al. 2019). Some, however, give more emphasis to urban amenities (Wenting 2011).

Despite the interest of this topic in general and its specific importance for French creative and cultural markets,^2^ empirical evidence for France is unfortunately still scarce. Notable exceptions are Sanchez-Serra (2014, 2013). Sanchez-Serra (2013) focuses on the clustering of creative clusters at travel-to-work areas (*Zones d’Emploi*) and identifies 63 artistic creative local labour systems, showing that creative employment is clearly more concentrated than is total employment, especially in and around big urban areas. Sanchez-Serra (2014) identifies creative clusters in France and their determinants, finding that the existence of information and communication technology jobs, education and the presence of foreign-born workers positively stimulate creative clustering. In the same line, Barois (2020) studies the link between the weight of creative and cultural activities in the territories and the attractiveness of the population showing that young workers and students prefer to locate in areas where the weight of the creative and cultural industries is important. Finally, although Chantelot (2010a) focuses on CI workforce rather than on firm entries, he identifies urban amenities and market opportunities as being among the main determinants of CI workforce concentration in large French urban areas.

## Methods

### Model specification

There is a degree of consensus that entry determinants are industry-specific (Audretsch and Fritsch 1999) and, more specifically, that CIs entries are affected by creativity-specific factors (see for instance, Coll-Martínez and Arauzo-Carod 2017; Sanchez-Serra 2016; Cruz and Teixeira 2014; Lazzeretti et al. 2012). Among these, the median household income (*income*) (the income elasticity of demand for cultural assets tends to be high) and higher levels of public investment in cultural issues (*public_investment*) should favour CIs location. Their location decision is also determined by residential amenities that in this paper are proxied by the following variables: the average number of days of sun (*sun*) and cumulative rain in mm (*rain*), that are expected to capture natural amenities, and the number of cinemas (*cinemas*) and museums (*museums*), that are expected to capture cultural amenities. Finally, areas that are more specialised in CIs (*LQ_creative*) should favour the entry of all kinds of firms because of the existence of knowledge spillovers in terms of creativity and innovation, as shown in Coll-Martínez and Arauzo-Carod (2017), and also should be more able to attract new firms because of the agglomeration advantages (localisation economies) created by the co-location of creative firms (Stam et al. 2008; De Jong et al. 2007; Lee et al. 2004; Scott 2000, 2006).

CIs also consider traditional location determinants (see Arauzo-Carod et al. 2010, for an extensive review). Among them, education (*human_capital*) and agglomeration economies (in this paper proxied by population density: *pop_density*) are important location factors whatever characteristics a firm may have. Share of manufacturing activities (*manufacturing*) is another well-known location determinant that fosters entries. Several different theories suggest that unemployment rates (*unemployment)* influence location decisions. Some studies show that high unemployment rates favour the creation of firms because of the lack of employment alternatives (Wagner and Sternberg 2004). However, other authors argue that high unemployment rates are linked to economic recession and, therefore, lower levels of consumption (Reynolds et al. 1994; Aubry et al. 2015) that in turn deter entries. Finally, geography and institutional issues matter (Guimarães et al. 2000), as firms need easy access to services provided in cores—hence, we need to control for distance to main cities such as Paris (*dist_paris)*. Moreover, proximity to the most important city of a country may capture, on the one hand, a potential competition effect in view of agglomeration of firms in the area and, on the other hand, a competitive advantage in terms of the services and amenities located in and around the city.

To analyse the determinants of CIs location decisions and their relationship with the CIs specialisation, we estimated the number of new establishments as a function of the specific local characteristics, in Eq. (1):

1 $$\begin{aligned} Firm\;entries_{it} = \beta_{0} + \beta_{1} human\_capital_{it} + \beta_{2} pop\_density_{it} + \beta_{3} income_{it} + \beta_{4} manufacturing_{it} + \beta_{5} unemployment_{it} + \beta_{6} public\_investment_{it} + \beta_{7} LQ\_creative_{it} + \beta_{8} dist\_paris_{it} + \beta_{9} rain_{it} + \beta_{10} sun_{it} + \beta_{11} cinema_{it} + \beta_{12} museums_{it} + u_{it} \\ \end{aligned}$$

where *Firm entries*_*it*_ is the number of firms located in area *i* across the period *t*. Our empirical strategy consists in estimating eight different models that share the same set of explanatory variables with different dependent variables (*Firm entries*_*it*_): all firms (*entry_t*), non-creative firms (*entry_noncrea*), creative firms (*entry_crea*), cinema and audiovisuals firms (*entry_audio*), sound recording (*entry_sound*), life performance (*entry_life*), arts craft (*entry_craft*), other music activities (*entry_other*), publishing firms (*entry_pub*), advertising firms (*entry_adv*) and videogames firms (*entry_videogames*). This strategy allows us to compare the location determinants of the group of firms considered, with particular focus on the impact of the specialisation in CIs.^3^

### Model selection

Most contributions in this field rely on cross section data, although a significant number use panel data approaches that cover a wide range of countries and entry typologies. Among them, for instance, we highlight the work of Hong et al. (2015) for Korea; Karahasan (2015) and Günalp and Cilasun (2006) for Turkey; Abdesselam et al. (2014) for France; Elert (2014) and Nyström (2007) for Sweden; Arauzo-Carod and Teruel-Carrizosa (2005) for Spain; Kangasharju (2000) for Finland, or Dunne et al. (1988) for the U.S. Using panel data offers some advantages over cross section data (Hsiao 2014). For instance, the possibility of introducing standard fixed effects in the regression potentially reduces the correlation effects of the explanatory variables with unobservables (which are difficult to control with cross section data). Thus, one of the main contributions of this paper is to provide evidence on CIs location determinants by using panel data.

Concretely, in this paper, we use Count Data Models to analyse the determinants of CIs location choices. The number of firm entries in a given region (in this paper, French departments) is a nonnegative integer (count) variable that is better estimated by techniques other than ordinary least squares (OLS) which can lead to biased, inefficient and inconsistent estimates (Long 1997).

Count Data Models (CDM) have commonly been used when dealing with the location phenomenon from a spatial point of view: i.e. when trying to explain how the local characteristics of different sites (e.g. municipalities, counties, regions) can influence firms’ decisions (Arauzo-Carod et al. 2010). These CDM include the Poisson Model (PM), the Negative Binomial Model (NBM), the Zero Inflated Poisson Model (ZIPM) and the Zero Inflated Negative Binomial Model (ZINBM). Although PM is the most popular CDM, it has two econometrical limits, “overdispersion” and “excess zeroes”. Since these problems may be solved using NBM, ZIPM and ZINBM, we follow Cameron and Trivedi (1998, 2005) in order to determine which of them is the most appropriate. To do this, we compute the following statistics: the Akaike Information Criterion (AIC), the Bayesian Information Criterion (BIC) and the Vuong test.

The descriptive statistics of the dependent variables in the firm entry model showed signs of overdispersion but, since there is at least one establishment (except for publishing and videogames industries)^4^ located in each department, there is not a zero-inflation problem. For this reason, we estimated a baseline specification using CDM and selected the specification with the best fit according to the above statistics. Tables 7 and 8 illustrate the results for these statistics and show that the NBM performed best according to AIC and BIC. The only exceptions are found for sound recording, publishing and videogames since the AIC, BIC and the Vuong test also favoured the ZINBM over the NBM. Nevertheless, the percentage of zeroes was not big enough to justify using an inflated model. Thus, we decided to use the NBM—for panel data and time fixed effects—for all the firm entry specifications, except for sound recording, publishing and videogames.

**Table 1:** Creative Industries firm entries by year

CIs Subgroups	2009	2010	2011	2012	2013
Cinema and audio-visuals	5292	5349	4925	5787	5697
*% in all the CIs*	*23%*	*23%*	*18%*	*25%*	*26%*
Sound recording	635	628	704	806	691
*% in all the CIs*	*3%*	*3%*	*3%*	*3%*	*3%*
Life performance	2892	2573	1951	2095	2421
*% in all the CIs*	*13%*	*11%*	*7%*	*9%*	*11%*
Arts Craft	2529	2153	1400	1246	1566
*% in all the CIs*	*11%*	*9%*	*5%*	*5%*	*7%*
Other artistic activities	6190	7113	6451	7446	6037
*% in all the CIs*	*27%*	*31%*	*23%*	*32%*	*28%*
Publishing	1329	1258	1373	1731	1578
*% in all the CIs*	*6%*	*6%*	*5%*	*7%*	*7%*
Advertising	3238	3315	10,506	3239	2895
*% in all the CIs*	*14%*	*14%*	*38%*	*14%*	*13%*
Videogames	489	477	513	791	815
*% in all the CIs*	*2%*	*2%*	*2%*	*3%*	*4%*
**All creative industries**	**22,594**	**22,866**	**27,823**	**23,141**	**21,700**
***% in all the economy***	***5%***	***5%***	***7%***	***6%***	***6%***
Non-CIs	399,068	412,649	373,909	387,695	367,551
*% in all the economy*	*95%*	*95%*	*93%*	*94%*	*94%*
All industries	421,662	435,515	401,732	410,836	389,251
	*100%*	*100%*	*100%*	*100%*	*100%*

*Source*: Authors, based on SIRENE data

**Table 2:** Creative Industries employment by year

CIs Subgroups	2009	2010	2011	2012	2013
Cinema and audio-visuals	4953	5076	4646	5357	5315
*% in all the CIs*	*11%*	*11%*	*10%*	*11%*	*11%*
Sound recording	3772	3529	3642	3775	3927
*% in all the CIs*	*8%*	*7%*	*8%*	*8%*	*8%*
Life performance	20,756	22,984	21,506	20,952	20,687
*% in all the CIs*	*46%*	*49%*	*46%*	*43%*	*43%*
Arts Craft	377	337	438	477	505
*% in all the CIs*	*1%*	*1%*	*1%*	*1%*	*1%*
Other music activities	10,332	10,669	11,838	12,803	13,299
*% in all the CIs*	*23%*	*23%*	*25%*	*26%*	*27%*
Publishing	1158	1125	1193	1568	1447
*% in all the CIs*	*3%*	*2%*	*3%*	*3%*	*3%*
Advertising	2944	3067	2765	2903	2608
*% in all the CIs*	*7%*	*7%*	*6%*	*6%*	*5%*
Videogames	374	391	432	746	748
*% in all the CIs*	*1%*	*1%*	*1%*	*2%*	*2%*
**All creative industries**	**44,666**	**47,178**	**46,460**	**48,581**	**48,536**
*% in all the economy*	*0.31%*	*0.34%*	*0.31%*	*0.32%*	*0.32%*
All industries	14,566,204	13,942,865	15,062,343	14,994,756	15,103,455
	*100%*	*100%*	*100%*	*100%*	*100%*

*Source*: Authors with INSEE data

As shown by many previous studies, neighbouring effects can be important. If the effects of the determinants of firm location decisions extend beyond departments, and this possible spatial dependence is not considered, then results may be biased and inconsistent. To account for spatial dependence, we also considered the spatially lagged variables of the independent variables (Spatial Lagged Model in the *X* (SLX)). These were estimated as: *W*_*Z* = *WZ,* where *Z* is a matrix that contains the independent variables and *W* is a row-standardised contiguity weighting matrix, an approach that has already been used in previous contributions for the case of French metropolitan *départements* (see for instance, Elhorst and Fréret 2009).^5^

## Data

### Data sources

All data in this paper relate to the 96 NUTS 3 departments of metropolitan France and include the location of new establishments (dependent variable) and a set of territorial characteristics (independent variables). The sources for the location of new establishments are the *Répertoire des Entreprises et des Établissemetns* (REE) and the *Système Informatique pour le Répertoire des Entreprises et de leurs Établissements* (SIRENE), supplied by the *Institut National de la Statistique et des Études Économiques* (INSEE). These sources provide comprehensive information on the location of establishments (both manufacturing and services) in France between 2009 and 2013, including geographical information (at regional and department levels), employment data, and other characteristics at the 4-digit NAF level. The local characteristics of French departments are taken from different sources such as INSEE, the French Government and Eurostat. Table 9 shows some descriptive statistics (see Table 10 for the main correlation results for these variables).

**Table 3:** Location determinants of firms (NB)

Dep. Var.:	(1)	(2)	(3)
Firm entries	All	Creative	Non-Creative
Human capital	0.023***	0.026***	0.023***
	(0.008)	(0.007)	(0.008)
pop_density	−8.70e − 05**	−6.33e − 05	−8.91e − 05**
	(4.16e − 05)	(5.25e − 05)	(4.10e − 05)
Income	1.27e − 05	1.55e − 05*	1.25e − 05
	(7.85e − 06)	(8.71e − 06)	(7.80e − 06)
Manufacturing	−1.152**	−1.585***	−1.132**
	(0.481)	(0.537)	(0.478)
Unemployment	4.604**	4.079*	4.636**
	(2.222)	(2.190)	(2.224)
Public investment	−0.001*	−0.00102*	−0.00109*
	(0.001)	(0.001)	(0.001)
LQ_creative	0.223*	0.286*	0.217
	(0.135)	(0.169)	(0.132)
dist_paris	−0.0003	−0.0006**	−0.0003
	(0.0003)	(0.0003)	(0.0003)
Rain	−0.0002	−0.0001	−0.0002
	(0.0002)	(0.0002)	(0.0002)
Sun	2.37e − 05	3.06e − 05	2.33e − 05
	(0.0001)	(9.69e − 05)	(0.0001)
Cinema	0.029***	0.029***	0.028***
	(0.005)	(0.005)	(0.005)
Museums	0.011**	0.011**	0.011**
	(0.005)	(0.005)	(0.005)
Constant	5.725***	2.514***	5.684***
	(0.606)	(0.614)	(0.606)
*N*	480	480	480
Departments	96	96	96
Time FE	*Y*	*Y*	*Y*
Wald *X*^*2*^	884.01	1124.97	854.26
Log pseudolikelihood	−3993.583	−2575.39	−3969.217
lnalpha	−2.452***	−2.393***	−2.452***
	(0.141)	(0.145)	(0.141)
Alpha	0.086	0.091	0.086
	(0.121)	(0.013)	(0.121)

Robust standard errors in parentheses, *** p < 0.01; **p  < 0.05; *p  < 0.1

**Table 4:** Location determinants of Creative Industries Subgroups (NB)

Dep. Var.:	(1)	(2)	(3)	(4)	(5)	(6)	(7)	(8)
Firm entries	Sound	Life	Craft	Other	Audio-visuals	Publishing	Advertising	Videogames
human capital	0.061***	0.026***	0.018***	0.0195***	0.033***	0.026***	0.031***	0.041***
	(0.0099)	(0.007)	(0.006)	(0.007)	(0.009)	(0.009)	(0.008)	(0.012)
pop_density	7.70e − 05	−5.12e − 05	−3.81e − 05	−7.48e − 05**	−7.55e − 05	2.67e − 05	−5.76e − 05	−2.45e − 05
	(7.66e − 05)	(4.48e − 05)	(2.72e − 05)	(3.08e − 05)	(6.00e − 05)	(6.62e − 05)	(4.14e − 05)	(3.14e − 05)
Income	3.34e − 05**	2.11e − 05**	1.54e − 05**	1.28e − 05*	2.28e − 05**	1.94e − 05**	2.38e − 05**	9.34e − 07
	(1.30e − 05)	(8.37e − 06)	(7.42e − 06)	(6.94e − 06)	(1.05e − 05)	(8.76e − 06)	(1.09e − 05)	(7.18e − 06)
manufacturing	−5.562***	−1.204*	−0.832	−1.142**	−2.310***	−3.429***	−2.045***	−4.863***
	(0.873)	(0.683)	(0.560)	(0.459)	(0.613)	(0.823)	(0.654)	(0.948)
Unemployment	−4.174	3.826*	5.010**	4.605**	3.583	−4.224	3.161	0.680
	(2.686)	(2.180)	(2.294)	(1.877)	(2.707)	(3.290)	(2.383)	(4.595)
public investment	0.0004	−0.0008	−2.04e − 05	−0.0006	−0.001	−0.0006	−0.001*	−0.0013
	(0.0009)	(0.0006)	(0.0006)	(0.0005)	(0.0007)	(0.0009)	(0.00067)	(0.00103)
LQ_$	−0.132	0.193*	0.0151	−0.00144	0.259*	0.0162	0.312**	0.505***
	(0.176)	(0.109)	(0.012)	(0.054)	(0.135)	(0.169)	(0.133)	(0.067)
Dist_paris	−0.0004	−0.001***	−0.001***	−0.007**	−0.0008**	−0.0006	−0.0003	0.0004
	(0.0005)	(0.0004)	(0.0003)	(0.0003)	(0.0004)	(0.0005)	(0.0004)	(0.0006)
Rain	−0.0001	−0.0001	−0.0003	−8.31e − 05	−0.0002	−0.0002	−0.0003**	−0.0003
	(0.0003)	(0.0008)	(0.0002)	(0.0001)	(0.0002)	(0.0002)	(0.0002)	(0.0003)
Sun	−9.38e − 05	0.0001	0.0001	2.74e − 05	−1.11e − 05	0.0002	−6.28e − 05	−0.0004*
	(0.0002)	(0.0001)	(0.0001)	(8.96e − 05)	(0.0001)	(0.0007)	(0.0001)	(0.0002)
Cinema	0.0120	0.0307***	0.0247***	0.0275***	0.0320***	0.0257***	0.0259***	0.0267***
	(0.007)	(0.005)	(0.005)	(0.005)	(0.006)	(0.007)	(0.006)	(0.008)
Museums	0.0076	0.0072	0.0142***	0.0116**	0.0098	0.0153**	0.0105*	0.0058
	(0.0078)	(0.0053)	(0.0041)	(0.0047)	(0.006)	(0.007)	(0.006)	(0.008)
Constant	−2.371***	0.166	0.773	1.816***	0.678	0.382	0.511	−1.108
	(0.873)	(0.658)	(0.632)	(0.598)	(0.693)	(0.787)	(0.724)	(1.088)
*Inflate*								
Pop	−0.000*	−	−	−	−	−0.000**	−	−0.000**
	(0.000)					(0.000)		(0.000)
constant	4.449***	−	−	–	−	2.463	−	3.12**
	(2.369)					1.642		(1.08)
*N*	480	480	480	480	480	480	480	480
Departments	96	96	96	96	96	96	96	96
Time FE	*Y*	*Y*	*Y*	*Y*	*Y*	*Y*	*Y*	*Y*
Wald *X*^*2*^	562.83	1044.05	1055.76	663.72	1061.69	505.00	2990.01	937.34
Log pseudolikelihood	−1088.507	−1620.841	−1525.022	−2060.629	−1950.449	−1385.312	−1872.121	−954.435
Lnalpha	−1.932***	−2.270	−2.402	−2.561	−2.159	−1.912	−2.096	−2.094***
	(0.185)	(0.165)	(0.165)	(0.129)	(0.159)	(0.194)	(0.161)	(0.432)
Alpha	0.146	0.103	0.090	0.077	0.115	0.148	0.123	0.123
	(0.027)	(0.017)	(0.015)	(0.010)	(0.018)	(0.029)	(0.019)	(0.053)
Vuong	3.51***	−	−	−	−	2.74**	−	4.09***

Robust standard errors in parentheses, ***p < 0.01; **p < 0.05; *p < 0.1

**Table 5:** Spatial Lag Model: Location determinants of firms (NB)

Dep. Var.:	(1)	(2)	(3)
Firm entries	All	Creative	Non-Creative
human capital	0.0224***	0.0256***	0.0222***
	(0.0027)	(0.0029)	(0.0027)
W_human capital	−0.0013	−0.0013	−0.0013
	(0.0012)	(0.0012)	(0.0012)
pop_density	−8.63e − 05***	−6.35e − 05***	−8.83e − 05***
	(1.20e − 05)	(1.27e − 05)	(1.20e − 05)
W_pop_density	−3.58e − 05*	−3.58e − 05*	−3.58e − 05*
	(2.18e − 05)	(2.18e − 05)	(2.18e − 05)
income	1.17e − 05**	1.42e − 05**	1.16e − 05**
	(5.43e − 06)	(5.72e − 06)	(5.42e − 06)
W_income	1.89e − 08	1.89e − 08	1.89e − 08
	(9.48e − 06)	(9.48e − 06)	(9.48e − 06)
Manufacturing	−1.163***	−1.593***	−1.143***
	(0.243)	(0.257)	(0.243)
W_manufacturing	0.366	0.366	0.366
	(0.457)	(0.457)	(0.457)
Unemployment	4.551***	3.939***	4.589***
	(0.906)	(0.963)	(0.906)
W_unemployment	−2.452	−2.452	−2.452
	(1.752)	(1.752)	(1.752)
public investment	−0.0011***	−0.0012***	−0.0011***
	(0.0002)	(0.0002)	(0.0002)
W_public investment	0.0005	0.0005	0.0005
	(0.0004)	(0.0004)	(0.0004)
LQ_creative	0.236***	0.306***	0.230***
	(0.058)	(0.059)	(0.058)
W_LQ_creative	0.146	0.146	0.146
	(0.128)	(0.128)	(0.128)
dist_paris	−0.0003**	−0.0006***	−0.0003**
	(0.0002)	(0.0001)	(0.0001)
Rain	−0.0002***	−0.0001*	−0.0002***
	(8.27e − 05)	(8.81e − 05)	(8.28e − 05)
Sun	1.98e − 05	2.67e − 05	1.93e − 05
	(5.36e − 05)	(5.64e − 05)	(5.36e − 05)
Cinema	0.0281***	0.0290***	0.0281***
	(0.0019)	(0.002)	(0.0019)
W_cinema	−0.0016	−0.0016	−0.0016
	(0.003)	(0.003)	(0.003)
Museums	0.0116***	0.0113***	0.0116***
	(0.0022)	(0.0023)	(0.0022)
W_museums	0.0033	0.0033	0.0033
	(0.0048)	(0.0048)	(0.0048)
Constant	5.768***	2.539***	5.720***
	(0.465)	(0.458)	(0.465)
*N*	480	480	480
Departments	96	96	96
Time FE	*Y*	*Y*	*Y*
Wald *X*^*2*^	885.42	995.95	877.11
Log pseudolikelihood	−3987.721	−2566.505	−3963.184

Robust standard errors in parentheses, ***p < 0.01; **p < 0.05; *p < 0.1

**Table 6:** Spatial Lag Model: Location determinants of Creative Industries Subgroups (NB)

Dep. Var: Firm entries	(1) Sound	(2) Life	(3) Craft	(4) Other	(5) Audio-visuals	(6) Publishing	(7) Advertising	(8) Videogames
human capital	0.0623***	0.0272***	0.0179***	0.0193***	0.0333***	0.0256***	0.0307***	0.0421***
	(0.0068)	(0.0039)	(0.0037)	(0.0029)	(0.0036)	(0.0054)	(0.0037)	(0.0078)
W_human capital	−0.0034	−0.0037**	−0.0044***	−0.0003	−0.0016	−0.0032	−0.0041**	−0.0029
	(0.0026)	(0.0016)	(0.0017)	(0.0013)	(0.0016)	(0.0022)	(0.0016)	(0.0027)
pop_density	6.92e − 05**	−4.91e − 05***	−3.59e − 05***	−7.17e − 05***	−7.68e − 05***	2.60e − 05	−5.46e − 05***	−2.26e − 05
	(3.22e − 05)	(1.42e − 05)	(1.10e − 05)	(9.50e − 06)	(1.73e − 05)	(2.17e − 05)	(1.23e − 05)	(1.77e − 05)
W_pop_density	−3.85e − 05	−2.16e − 05	−1.46e − 05	−2.74e − 05*	−1.85e − 05	−5.85e − 06	−1.91e − 05	2.96e − 05
	(6.98e − 05)	(2.40e − 05)	(1.92e − 05)	(1.57e − 05)	(2.96e − 05)	(4.62e − 05)	(2.13e − 05)	(2.99e − 05)
Income	3.01e − 05***	1.83e − 05***	1.50e − 05**	1.13e − 05**	2.08e − 05***	1.91e − 05**	2.29e − 05***	−2.64e − 07
	(9.18e − 06)	(6.35e − 06)	(6.13e − 06)	(5.24e − 06)	(6.76e − 06)	(8.34e − 06)	(7.07e − 06)	(9.03e − 06)
W_income	−1.54e − 05	−1.17e − 07	−6.41e − 06	9.81e − 06	2.59e − 06	3.49e − 06	−9.09e − 06	5.10e − 06
	(1.99e − 05)	(1.28e − 05)	(1.29e − 05)	(9.79e − 06)	(1.21e − 05)	(1.67e − 05)	(1.26e − 05)	(2.22e − 05)
Manufacturing	−5.126***	−1.098***	−0.914***	−1.130***	−2.356***	−3.475***	−1.984***	−4.993***
	(0.563)	(0.355)	(0.331)	(0.258)	(0.318)	(0.457)	(0.334)	(0.651)
W_manufacturing	2.629**	1.149*	0.655	0.315	0.204	1.693**	1.330**	−0.441
	(1.022)	(0.645)	(0.623)	(0.490)	(0.606)	(0.836)	(0.640)	(1.121)
Unemployment	−4.293**	3.577***	4.776***	4.591***	3.228***	−4.725***	2.697**	−0.0459
	(1.945)	(1.235)	(1.246)	(0.958)	(1.194)	(1.719)	(1.240)	(2.358)
W_unemployment	4.623	0.903	−1.156	−2.855	−1.529	−0.998	−3.198	1.376
	(3.614)	(2.295)	(2.362)	(1.834)	(2.296)	(3.122)	(2.365)	(3.981)
public investment	0.0003	−0.001***	−3.49e − 05	−0.0006***	−0.001***	−0.0006	−0.0014***	−0.0012*
	(0.0005)	(0.0003)	(0.0003)	(0.0002)	(0.0003)	(0.0004)	(0.0003)	(0.0006)
W_public investment	0.0016*	0.0013**	0.0007	0.0007*	0.0008*	0.0005	0.0011**	0.0012
	(0.0008)	(0.0005)	(0.0005)	(0.0004)	(0.0005)	(0.0007)	(0.0005)	(0.0009)
LQ_$	−0.104	0.205***	0.0170	−0.00914	0.271***	0.0357	0.335***	0.527***
	(0.0897)	(0.0668)	(0.0114)	(0.0280)	(0.0527)	(0.0713)	(0.0570)	(0.0484)
W_LQ_$	0.231	−0.0348	0.0109	−0.0193	0.0402	0.0642	0.277**	−0.0145
	(0.213)	(0.127)	(0.0201)	(0.0502)	(0.105)	(0.189)	(0.126)	(0.142)
dist_paris	−0.0004	−0.0009***	−0.0009***	−0.0007***	−0.0008***	−0.0006***	−0.0002	0.0004
	(0.0003)	(0.0001)	(0.0002)	(0.0001)	(0.0002)	(0.0002)	(0.0002)	(0.0003)
Rain	−0.0002	−0.0002	−0.0003**	−8.42e − 05	−0.0002*	−0.0002	−0.0004***	−0.0004**
	(0.0002)	(0.0001)	(0.0001)	(8.70e − 05)	(0.0001)	(0.0002)	(0.0001)	(0.0002)
Sun	−6.04e − 05	0.0001*	0.0001	3.16e − 05	−8.47e − 06	0.0002*	−5.59e − 05	−0.0004***
	(0.0001)	(7.33e − 05)	(7.29e − 05)	(5.59e − 05)	(7.01e − 05)	(0.0001)	(7.21e − 05)	(0.0001)
Cinema	0.0141***	0.0305***	0.0240***	0.0272***	0.0318***	0.0253***	0.0254***	0.0265***
	(0.004)	(0.002)	(0.002)	(0.002)	(0.0024)	(0.0033)	(0.0024)	(0.004)
W_cinema	0.0068	0.001	0.0013	−0.0036	0.0003	0.0069	−0.0021	0.0037
	(0.0072)	(0.0045)	(0.0045)	(0.0035)	(0.0043)	(0.006)	(0.0046)	(0.0081)
Museums	0.0062	0.0078***	0.0143***	0.0124***	0.0099***	0.0138***	0.0107***	0.0034
	(0.0044)	(0.0028)	(0.0028)	(0.0023)	(0.0028)	(0.0037)	(0.0029)	(0.0042)
W_museums	−0.0149	0.0044	0.0039	0.0095*	0.0012	−0.0168*	−0.0016	−0.0219*
	(0.0105)	(0.0066)	(0.0065)	(0.0051)	(0.0064)	(0.0089)	(0.007)	(0.0115)
Constant	−3.362***	−0.470	1.425**	1.667***	0.981	0.584	0.401	0.005
	(1.054)	(0.651)	(0.626)	(0.492)	(0.603)	(0.852)	(0.638)	(1.173)
*Inflate*								
Ppop	−0.000**	−	−	−	−	−0.000***	−	−0.000***
	(0.000)					(0.000)		(0.000)
constant	4.506	−	−	−	−	2.275**	−	2.83***
	(1.995)					(0.979)		(0.721)
*N*	480	480	480	480	480	480	480	480
Time FE	*Y*	*Y*	*Y*	*Y*	*Y*	*Y*	*Y*	*Y*
Log pseudolikelihood	−1075.408	−1610.265	−1517.873	−2053.83	−1946.125	−1378.752	−1859.683	−953.819

Robust standard errors in parentheses; ***p < 0.01; **p < 0.05; *p < 0.1

Regarding the definition of CIs, we use the APUR-INSEE proposal (2014) as it is the official classification of CIs used in France and roughly relies on the UNCTAD’s (2008) proposal, which is the most widely accepted by researchers (see, among others, Lazzeretti et al. 2012; Coll-Martínez and Arauzo-Carod 2017). According to this criterion, we include 29 sectors in CIs, classified into 8 subgroups (cinema and audio-visuals, sound recording, life performance, arts craft, other music activities, publishing, advertising and videogames (see NAF-Rev. 2 industry classification in Table 11).^6^ Table 1 illustrates the 2009‒2013 period showing an increasing trend (between 2009 and 2011) followed by a short period of attrition that fits with the economic trend of these years, and Table 2 shows a weak decrease in employment in CIs sectors during the same period. The choice of the time span is driven by data availability at the level of NUTS 3 regions. Nevertheless, it is worth underlying that the considered period in our analysis starts after the Global Financial Crisis that hit France and its regions in 2007–2008, lowering then potential bias due to market turbulence. Hence, we analyse the location attributes explaining the entry of firms in creative industries in a context of a certain economic recovery (Sensier et al. 2016; Arcuri et al. 2019) as the economy has moved from negative growth rates (in 2009) to positive ones (over 2010–2013).

### Stylised facts about creative industries and firm location in French Departments

**Fig. 1:**
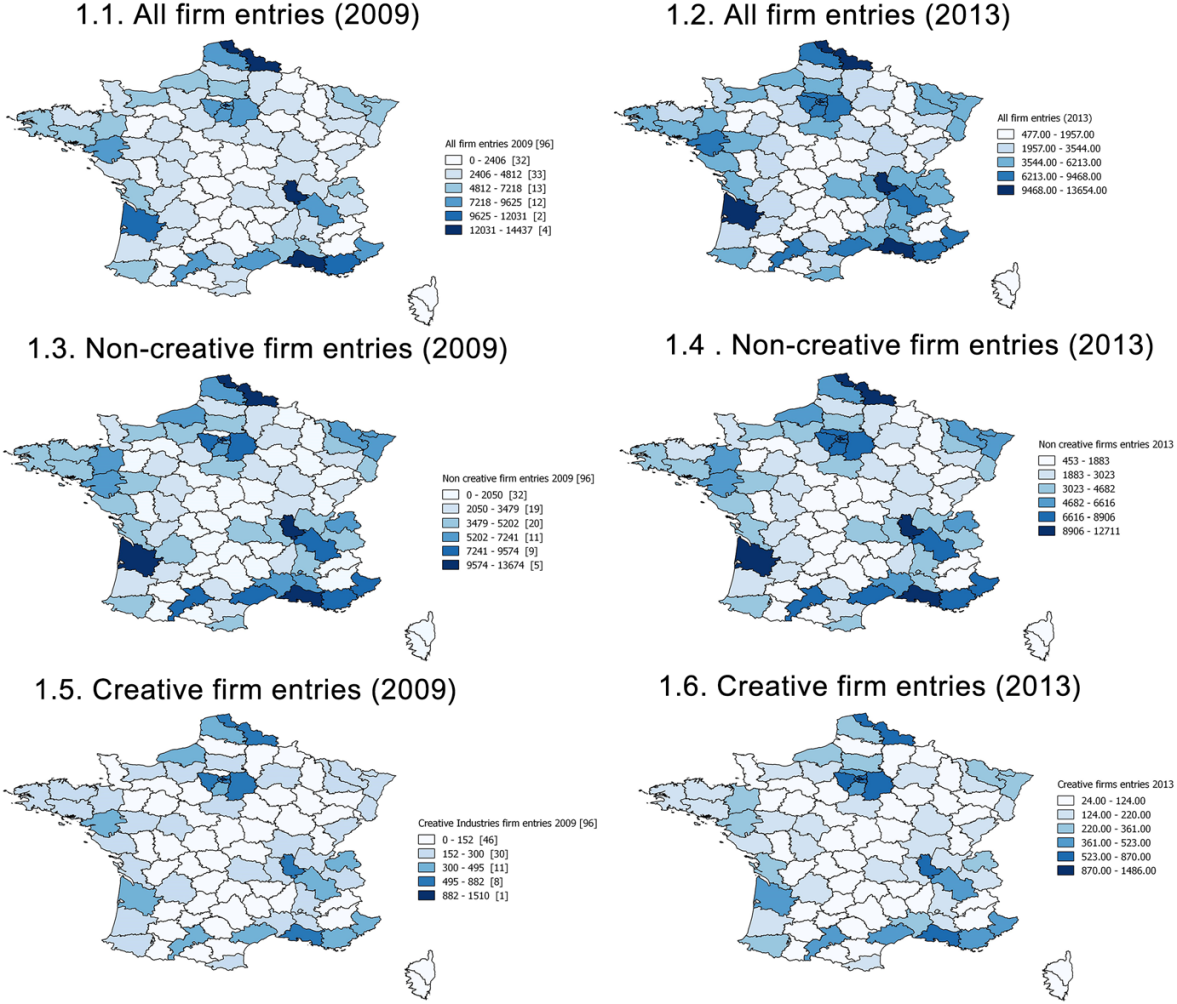
Firm entries by department. Source: Authors with SIRENE data

Figure 1 compares the location patterns of all firms, non-creative and creative firms. For both years (2009 and 2013), roughly 75% of all firms locate in and around Île-de-France and in the most populated departments such as Nord, Rhône, Bouches-sur-Rhône or Gironde, the same areas where most cultural jobs are located (Cléron and Patureau 2007). Thus, it seems clear that one of the key determinants of a firm’s location decision is the attractiveness of these densely populated areas, specialised labour markets, availability of suppliers and knowledge spillovers.

Similar spatial distributions hold for both creative and non-creative firms. Moreover, it has not significantly changed from 2009 to 2013. Although the number of new creative firms has increased over these years, they have kept the same agglomeration pattern around larger cities,^7^ as it has been demonstrated by other studies using spatial analysis tools (Chantelot et al. 2010a, b).

In order to identify location patterns for CIs in French departments, we calculate a Location Quotient (LQ), using employment data (*Effectif salarié déclaré par les établissements*) taken from INSEE. The same index has been used by other scholars but with different specifications (for example, in Lazzeretti et al. 2012). This index compares the relative specialisation of a department in a sector in relation to the national (France) average and is defined as:

$${\text{LQ}}\_creative_{ij} = (L_{ij} /L_{j} ) / (L_{i} {/}L)$$

where *L*_*ij*_ is the workforce in the creative industry *j* in department *i*, *L*_*j*_ is the total workforce in the creative industry *j, L*_*i*_ is the total workforce in department *i*, and *L* is total employment in the area (France). An LQ greater than 1 indicates that the clustering of a creative industry *j* in department *i* is larger than the national average, hence the department is specialised in CIs.

**Fig. 2:**
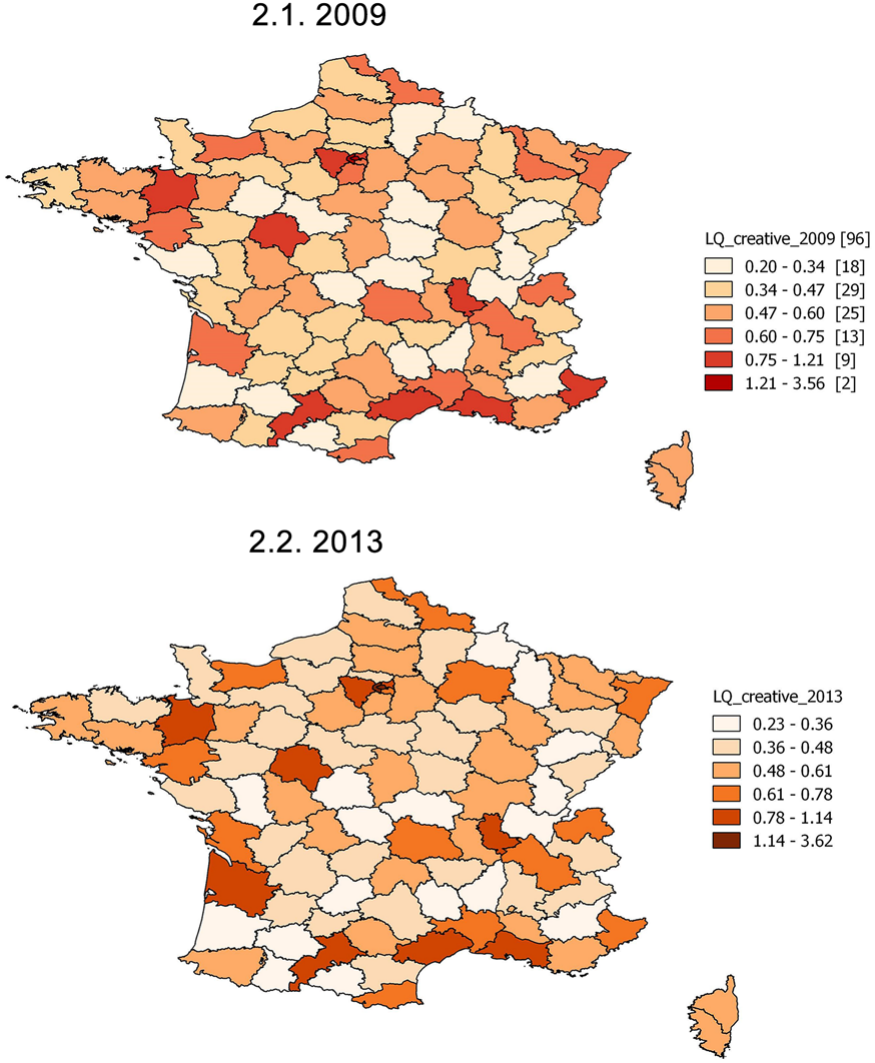
Specialisation in creative industries by department. Source: Authors with INSEE data

Figure 2 shows LQ results for the ten most specialised French departments and the spatial distribution of LQ in CIs for 2009 and 2013, respectively. Departments located in the Île-de-France region are the most specialised in CIs (all with values higher than 1). Concretely, Hauts-de-Seine and Paris departments stand out with a LQ greater than 3 for both years. Although, since they have values below 1, the remaining most populated departments are not specialised in CI’s, nevertheless they comprise most of the creative employment in France. These results have not significantly changed over these years.

## Results

### Main results

Table 3 shows the results of the econometric estimation of CIs location determinants. Negative Binomial estimates are presented for all firm entries, both creative and non-creative, in order to compare the determinants of location decisions of different types of industries. In general, for all types of entries, most of the explanatory variables are significant, but there are some remarkable differences between the creative and non-creative industries. Specifically, population density (a proxy for agglomeration economies) has a negative effect over all industries and non-creative industries, although the coefficient is not significant for CIs.^8^ Nevertheless, the role of population density is not clear, as correlation analysis shows a significant and positive relationship with all entries, but especially with those of CIs. This result could be understood in terms of an unknown relationship between the location quotient of CIs and population density, although the influence of density over entries seems to be blurred by other explanatory variables. The aggregated income level of departments also plays a different role as it only boosts the entries of CIs. This may suggest some structural differences in terms of markets: CIs may, for example, target the upper income levels of population. In a similar way, specialisation in CIs (*LQ_creative*) pushes up entries in CIs and for all firms but has no significant effect on entries of non-creative firms. This result supports our assumption regarding the positive effects of specialisation in CIs in terms of attracting new economic activity, no matter what the industry of the entering firms. Noticeably, departments specialised in CIs are more likely to attract new businesses. In terms of geographical position, a greater distance to Paris deters the entry of creative firms, as they may have more difficulties in establishing networking activities and an access to cultural amenities (that are highly concentrated in the French capital).^9^

Despite specific effects at industry level, there are common location determinants that act in a similar way across different types of industries (i.e. creative and non-creative), similarly to previous results also for Catalonia by Arauzo-Carod (2021) and Coll-Martínez and Arauzo-Carod (2017). In this sense, entries in all subgroups are attracted to areas that have more people enrolled in education (this is a necessary production factor, no matter the industry), while they are repelled from areas with more manufacturing activity. This result may be explained by the fact that these areas are associated with negative externalities that do not fit with cultural and creative environments. Surprisingly, regional economic conditions favour areas with high unemployment rates and, similarly, those that receive higher levels of public investment. Cultural amenities (i.e. cinema and museums), exert the same positive effect on entries across all firm profiles whilst climate conditions (rain and sun) have no significant effect on entries. Nevertheless, it is important to precise that our approach analyses location determinants for both creative and non-creative industries considering firms included in these categories, but without taking into account the profile of workforce at firm/industry level. In this sense, creative and non-creative jobs coexist in both creative-and-non-creative firms, although with different shares.^10^

Negative Binomial estimates are presented in Table 4 for entering firms belonging to sound, life, craft, other, audio-visuals, publishing, advertising, and videogames, in order to compare the determinants of location decision for these CIs. This strategy allows us to analyse the location behaviour of specific CIs, given that overall results may not reveal some heterogeneities due to the locational specificities of each CIs.

As expected, many CIs subgroups share most of the location determinants, such as the positive role played by human capital, income and the cultural amenities (i.e. museums and cinema), as well as the negative effect of share of manufacturing activity, but there are noticeable differences for other determinants. In particular, we may distinguish between (mostly) cultural oriented and (mostly) technology-oriented subgroups: the former includes arts, sound, life, craft, other activities and publishing, whilst the latter include audio-visuals, advertising and videogames. Although the patterns are not clearly divided into two groups,^11^ the main difference is found in the role played by localisation economies since, while for technology-oriented subgroups, location quotients in their subgroup foster entries, this effect is only found for one out of the six cultural oriented subgroups, that is, life performing arts. This is a relevant issue, since agglomeration economies at department level matter for these activities, although it could also be argued that their geographical scope is much smaller than that of a department. Surprisingly, except for Audio-visuals, technology-oriented subgroups do not suffer from distance to Paris, suggesting that it is possible to attract such firms outside the Île-de-France region.^12^

Regarding unemployment rates, technology-oriented subgroups also have some specificities as they are not positively attracted by them, as for the rest of subgroups. This may be explained by the fact that, for these industries, the creation of new firms is mainly driven by innovative ideas or market opportunities. Thus, the conditions leading to higher unemployment rates may deter innovative-based CIs entries (Storey 1991; Fritsch 2008). For the cultural CIs subgroups (i.e. life performance, arts craft and other artistic activities) the impact of unemployment is positive and significant. This is consistent with the findings of Aubry et al. (2015) who show that start-ups in France are mainly explained by a refugee effect (i.e. the creation of firms is a strategy to escape from unemployment). This result is also in line with the higher part-time work and unemployment rates that usually characterise employment in the more artistic and cultural CIs (Faggian et al. 2013; Pareja-Eastaway 2016).

In order to account for inter-department neighbouring externalities for both the entries grouped (Table 5) and at a subgroup level (Table 6), we estimate an enlarged location decision model including such spatial externalities.

Regarding Table 5, almost all the key location determinants remain significant as in previous estimations. However, by adding spatial lagged variables, some variables such as population density, income, distance to Paris and rain levels become significant. Population density and income tend to be closely linked to market strength, which is a key location factor, as well as that increased distance to main economic centres usually having a negative effect due to lower attractiveness of these areas (Coll-Martínez and Arauzo-Carod 2017).

The effects of the specialisation in creative industries remain significant at the department level, but they do not seem significant beyond department borders. In other words, creative firms seem to be only affected by specialisation in CIs in the departments where they locate, but not by surrounding areas. This is a quite reasonable result as the spatial scope of agglomeration externalities captured by LQ_creative tends to diminish after very short distances, as reported previously by Coll-Martínez et al. (2019) for Barcelona’s neighbourhoods, Cruz and Teixeira (2014) for Portuguese municipalities, and Wojan et al. (2007) for US counties.

Finally, subgroup estimation including spatial lags (Table 6) slightly modifies the previous findings when taking spatial effects into account. In particular, the negative effects of population density and public investments on entries now become significant for most of the subgroups. A noticeable exception is Videogames since, for this industry, population density does not deter entries. This result fits perfectly with the existing literature regarding the locational patterns of Videogames industry, as empirical evidence has demonstrated the strong urban-core preferences of firms belonging to that industry (Moriset 2003; Méndez-Ortega and Arauzo-Carod 2019, 2020).

In general terms, the subgroup estimations including spatial lags increase the significance of most of covariates used in previous estimations, although failing to identify strong significant influences of neighbouring departments. This result may be, to some extent, explained by the fact that neighbouring relations have been defined by considering a spatial contiguity matrix. Still, the use of a spatial contiguity matrix provides the best fit of the model. Anyway, the inclusion of spatial lags allows us to capture any source of spatial dependence in terms of knowledge spillovers spreading beyond geographical limits and that cannot be considered otherwise.

## Conclusion

In this paper, we estimate the location determinants of new creative industries (CIs) firms across metropolitan France departments over the period 2009–2013. The econometric results show that the location determinants of creative and non-creative firms are quite similar, and that both creative and non-creative firms are positively affected by the specialisation in CIs. This influence supports public investments in these industries in view of the positive externalities arising from their spatial concentration on firm entry. Our results also show that there are some locational specificities among CIs activities due to their heterogeneity. Finally, when accounting for spatial dependence, we found that creative firms seem to be only affected by CI specialisation in the departments where they locate, but not in surrounding areas, so the spatial scope of effects is less than a standard department.

Our results are in line with those of previous empirical contributions and support the positive association between the concentration of creative workers and new firms’ creation at the department level (Scott 2006; Lee et al. 2004; Stam et al. 2008; Audretsch and Belitski 2013; Coll-Martínez and Arauzo-Carod 2017). Moreover, they are in line with previous findings highlighting the uneven geographic distribution of creative people, mainly concentrated in Paris and larger French cities (Chantelot 2010a, 2010b; Sanchez-Serra 2013, 2014). Consequently, our results help to fill a gap in the empirical literature in terms of a lack of knowledge of the processes driving the entry decisions of CIs firms.

This paper has certain limitations. Since it is focused on location determinants of CIs at a quite aggregated level, it remains for future research to analyse whether our results hold for alternative geographical disaggregation levels such as municipalities or metropolitan areas. Some empirical evidence already exists for the French case (see Chantelot 2010a) and this indicates that there are differences between big and medium-small urban areas. Additionally, due to the huge concentration of CIs in Paris and the municipalities in its metropolitan area (see Boix et al. 2016), it would be advisable to carry out a detailed and spatially disaggregated analysis for this region. Future efforts will also be conducted to understand and identify the complexity and the cross-fertilisation of different creative jobs working in other industries than the CIs (Bakhshi and McVittie 2009; Cerisola 2018a, b; Innocenti and Lazzeretti 2019).

Policy implications from our results point to the importance of achieving a critical mass of creative activities as a necessary condition for attracting firm entries from these industries. However, from a territorial cohesion point of view, is not a desirable to reinforce excessive concentration of CIs in and around main urban areas. Thus, given (i) the uneven spatial distribution of CIs entries in French departments and (ii) the fact that the most populated departments (where most CIs locate) are the ones receiving the most public funding and support for cultural and creative activities, less populated (rural) areas might benefit little from the potential of CIs for economic development and sustainable growth. Thus, there is room for policy interventions which can support CIs in these (peripheral) areas. In fact, the COVID-19 pandemic and its immediate consequences (i.e. shutdowns, economic slowdown, physical distance) may impact the activity and location choices of CIs firms. At the same time, this crisis may provide an opportunity for less urbanised areas to attract the creation of creative firms. We however leave this interesting and promising approaches for further research.

## Appendix

See Tables 7, 8, 9, 10 and 11.

**Table 7:** Selection model tests

Model 1 (All firms)	AIC	BIC	Vuong test
Poisson	151,752.47	151,823.42	−
Negative binomial	8,023.16	8,023.16	−
Zero-inflated Poisson	151,756.47	151,756.47	−
Zero-inflated negative binomial	8027.165	8110.64	−
Model 2 (Creative)	AIC	BIC	Vuong test
Poisson	12,696.39	12,767.79	−
Negative binomial	5,245.14	5,245.14	−
Zero-inflated Poisson	12,700.79	12,780.09	−
Zero-inflated negative binomial	5.249.14	6165.74	−1.72
Model 3 (Non-Creative)	AIC	BIC	Vuong test
Poisson	163,629.84	163,708.25	−
Negative binomial	9486.6961	9,569.4622	−
Zero-inflated Poisson	163,629.84	163,708.25	−
Zero-inflated negative binomial	9,490.6961	9582.1744	−

****p* < 0.01; ***p* < 0.05; **p* < 0.1

**Table 8:** Selection model’s tests for Creative Industries Subgroups

Model 1 (Sound)	AIC	BIC	Vuong test
Poisson	2580.85	2651.81	−
Negative binomial	2285.78	2360.91	−
Zero-inflated Poisson	2431.36	2510.66	3.27***
Zero-inflated negative binomial	2217.01	2,300.49	3.51***
Model 2 (Life)	AIC	BIC	Vuong test
Poisson	3916.10	3987.05	−
Negative binomial	3277.68	3352.81	−
Zero-inflated Poisson	3914.82	3994.12	0.77
Zero-inflated negative binomial	3281.68	3365.16	0
Model 3 (Craft)	AIC	BIC	Vuong test
Poisson	3414.11	3485.06	−
Negative binomial	3086.04	3161.17	−
Zero-inflated Poisson	3415.13	3494.43	0.66
Zero-inflated negative binomial	3090.04	3173.52	0
Model 4 (Other)	AIC	BIC	Vuong test
Poisson	5635.51	4157.26	−
Negative binomial	5706.47	4232.39	−
Zero-inflated Poisson	−	−	−
Zero-inflated negative binomial	−	−	−
Model 5 (Audio-visuals)	AIC	BIC	Vuong test
Poisson	6503.13	6581.54	−
Negative binomial	4559.39	4642.15	−
Zero-inflated Poisson	6495.85	6582.97	0.91
Zero-inflated negative binomial	4563.39	4654.86	−0.88
Model 6 (Publishing)	AIC	BIC	Vuong test
Poisson	3,974.44	4,052.85	−
Negative binomial	3,360.88	3,443.65	−
Zero-inflated Poisson	3,816.52	3,903.64	3.79***
Zero-inflated negative binomial	3,308.36	3,399.84	2.84**
Model 7 (Advertising)	AIC	BIC	Vuong test
Poisson	6011.27	6089.68	−
Negative binomial	4415.87	4498.64	−
Zero-inflated Poisson	5981.14	6068.26	0.61
Zero-inflated negative binomial	4404.32	4495.80	0.5
Model 8 (Videogames)	AIC	BIC	Vuong test
Poisson	2508.96	2587.37	−
Negative binomial	2377.21	2459.98	−
Zero-inflated Poisson	2339.90	2427.03	4.78***
Zero-inflated negative binomial	2280.41	2371.89	4.67***

****p* < 0.01; ***p* < 0.05; **p* < 0.1

**Table 9:** Summary statistics

Variable	Description	Source	Obs	Mean	Std. Dev	Min	Max
entry_t	Number of total firm entries	Own elaboration with INSEE	480	4251.9	3125.7	477.0	14,608.0
entry_crea	Number of creative firm entries	Own elaboration with INSEE	480	228.0	222.9	22.0	1623.0
entry_noncrea	Number of non-creative firm entries	Own elaboration with INSEE	480	4027.7	2925.2	453.0	13,849.0
entry_sound	Number of sound recording firm entries	Own elaboration with INSEE	480	6.8	11.7	0.0	96.0
entry_life	Number of life performance firm entries	Own elaboration with INSEE	480	25.7	25.1	0.0	166.0
entry_craft	Number of arts craft firm entries	Own elaboration with INSEE	480	17.9	15.3	0.0	83.0
entry_other	Number of other music and arts firm entries	Own elaboration with INSEE	480	66.6	45.6	6.0	264.0
entry_audio	Number of audio-visual firm entries	Own elaboration with INSEE	480	57.3	68.5	1.0	545.0
entry_pub	Number of publishing firm entries	Own elaboration with INSEE	480	14.7	30.8	0.0	322.0
entry_adv	Number of advertising firm entries	Own elaboration with INSEE	480	44.9	54.5	0.0	472.0
entry_videogames	Number of videogames firm entries	Own elaboration with INSEE	480	6.3	13.3	0.0	123.0
Human capital	Number of secondary students for 1000 inhabitants	http://www.collectivites-locales.gouv.fr/	480	82.1	7.4	61.8	99.8
pop_density	Population per squared km on 1st January	Eurostat	480	559.7	2454.0	14.8	21,347.0
Income	Disposable income in €/inhabitant	http://www.collectivites-locales.gouv.fr/	480	12,820.6	4912.0	821.1	53,829.0
Manufacturing	Manufacturing employment rate	Own elaboration with INSEE	480	0.2	0.1	0.0	0.4
Unemployment	Unemployment rate	http://www.collectivites-locales.gouv.fr/	480	0.1	0.0	0.0	0.2
Public investment	Actual investment expenditure in € / inhab	http://www.collectivites-locales.gouv.fr/	480	238.9	79.7	66.8	666.6
LQ_creative	Location Quotient in Creative Industries	Own elaboration with INSEE	480	0.6	0.5	0.2	3.7
LQ_sound	Location Quotient in Sound Recording	Own elaboration with INSEE	480	0.3	0.8	0.0	7.5
LQ_life	Location Quotient in Life Performance	Own elaboration with INSEE	480	0.6	0.5	0.0	4.1
LQ_craft	Location Quotient in Arts Craft	Own elaboration with INSEE	480	0.9	1.8	0.0	13.2
LQ_other	Location Quotient in Other Music and Arts Activities	Own elaboration with INSEE	480	1.1	0.6	0.1	3.9
LQ_audio	Location Quotient in Audio-visual	Own elaboration with INSEE	480	0.5	0.6	0.1	4.8
LQ_pub	Location Quotient in Publishing	Own elaboration with INSEE	480	0.5	0.6	0.1	5.4
LQ_adv	Location Quotient in Advertising	Own elaboration with INSEE	480	0.6	0.4	0.0	3.9
LQ_videogames	Location Quotient in Videogames	Own elaboration with INSEE	480	0.5	0.7	0.0	4.2
dist_paris	Distance in km from the capital of Department to Paris	Own elaboration	480	353.5	205.5	0	918.9
Rain	Cumulate rain in a year in mm	Eider—French Government	480	800.5	204.5	423.2	1625.3
Sun	Cumulate sunny time in hours	Eider—French Government	570	1973.2	385.3	73.1	3058.0
Cinema	Number of cinemas	CNC Eider—French Government	480	21.2	14.1	3.0	88.0
Museums	Number of museums	INSEE	480	12.7	8.7	2.0	59.0

*Source*: Authors

**Table 10:** Correlation of main explanatory variables

	1	2	3	4	5	6	7	8	9	10	11	12
1. Human capital	1											
2. Pop_density	0.1255*	1										
3. Income	0.3447*	0.4078*	1									
4. Manufacturing	−0.1804*	−0.3672*	−0.2838*	1								
5. Unemployment	0.1233*	−0.0707	0.0289	−0.1363*	1							
6. Public investment	−0.1934*	−0.1887*	−0.3543*	−0.0776	−0.1754*	1						
7. LQ_creative	0.2000*	0.8446*	0.4591*	−0.4485*	−0.0771	−0.1566*	1					
8. dist_paris	−0.2999*	−0.3144*	−0.1670*	−0.2824*	0.1562*	0.3689*	−0.2359*	1				
9. Rain	−0.0936*	−0.1638*	0.0197	0.2155*	−0.0563	0.0051	−0.2242*	0.2175*	1			
10. Sun	−0.1187*	−0.1281*	−0.0182	−0.4306*	0.2231*	0.3041*	−0.0213	0.6739*	−0.1226*	1		
11. Cinema	0.5033*	0.5203*	0.4242*	−0.4691*	−0.0765	−0.1715*	0.5744*	0.0112	0.0235	0.1002*	1	
12. Museums	0.3500*	0.4692*	0.3542*	−0.2476*	0.1219*	−0.2563*	0.5117*	−0.0975*	−0.1122*	0.0597	0.6372*	1

*Source*: Authors. Significance level: **p* < 0.05

**Table 11:** Creative Industries Classification

CIs subgroups	Sectors	Code APE-NAF Rev. 2
Cinema & Audio-visuals (*audiovisuals*)	Reproduction of sound recording	1820Z
	Production of films and shows for television	5911A
	Production of institutional and advertising films	5911B
	Production of film for cinema	5911C
	Post-production of films and shows for television	5912Z
	Distribution of cinematographic films	5913A
	Editing and distribution of videotapes	5913B
	Projection of cinematographic films	5914Z
	Broadcasting and distribution of radio shows	6010Z
	Broadcasting of generalist channels	6020A
	Broadcasting of theme channels	6020B
	Photographic activities	7420Z
Sound recording (*sound*)	Sound recording and music editing	5920Z
Life performance (*life*)	Life performing arts	9001Z
	Life performing arts supporting activities	9002Z
Arts craft (*craft*)	Arts and crafts artistic creation	9003A
Other artistic activities (*other)*	Other activities related to artistic creation	9003B
	Other activities related to entertainment	9329Z
Publishing (*publishing*)	Publishing of books	511Z
	Publishing of newspapers	5813Z
	Magazine publishing	5814Z
	Other publishing activities	5819Z
	Other news agencies activities	6391Z
Advertising (*advertising*)	Advertising agencies activities	7311Z
	Management of advertising media	7312Z
Videogames (*videogames*)	Publishing of videogames	5821A
	Publishing of software systems	5829A
	Publishing of software for development tools and languages	5829B
	Publishing applicative software	5829C

*Source*: Authors following APUR-INSEE classification

## Footnotes

1 The list of specific activities included among CIs is quite wide but, in general terms, the following are considered: Arts, Advertising, Cinema, Fashion, Publishing, R&D, and Software. See Sect. 4 (Data) for details and Table 9 for the complete list of industries.

2 See Chantelot (2010b) for an analysis of French creative class in terms of workforce.

3 Specifically, zeroes were 6.94% for publishing and 30% for videogames entries.

4 The key variable LQ_creative is replaced in each model for a LQ in each subgroup of CIs, that is, LQ_audio, LQ_sound, LQ_life, LQ_craft, LQ_other, LQ_pub, LQ_adv and LQ_videogames.

5 Other spatial weighting definitions were considered such as 5 nearest neighbours or an inverse distance-based matrix. Even so, the best fit of the model is obtained when we rely on a row-standardised contiguity weighting matrix, an approach that has already been used in previous contributions for the case of French metropolitan *départements* (see for instance, Elhorst and Fréret 2009).

6 Although the Architecture and Engineering industries are typically included among CIs, we decided to exclude them as they have a very particular location patterns, are concentrated in some departments, and include an important share of nuclear activities (noticeably in Territoire Belfort where there is a cross-border cluster on both sides of the French-Swiss border) which are far away from CIs.

7 See Julien (2002) for empirical evidence regarding France.

8 Chantelot (2010a) analyses determinants of CIs workforce location and reaches a similar result for big urban areas in France.

9 Additionally, we tested alternative covariates for proxying proximity to the political power (regional capital), climate conditions (temperature and humidity), natural amenities (coast, forest area and natural parks), tourism activities (lodging size), and diversity (foreign population), but model fit did not improve when they were included, and main results generally remained quite similar.

10 Although the focus of this paper is on creative industries and not on creative jobs, which demands a different type of dataset, there is empirical evidence showing that creative jobs exert positive effects over close local service employment (Gutierrez-Posada et al. 2021).

11 We have to take into account that the way in which these activities have been grouped also differs. Concretely, CIs subgroups include NACE5 subsectors but with different quantities (see Table 11): Craft and Sound include 1 subsector; Life, Advertising and Other include 2 subsectors; Publishing and Videogames include 5 subsectors; and Audio-visuals includes 12 subsectors.

12 These results could be also affected by firm size, however information of the size of firms was not available. In any case, firms in cultural and creative industries tend to be of smaller size than the rest of economic activities (i.e. size variation is lower inside them) and, partially due to that reason, papers focusing on entry determinants or spatial distribution of firms use to group firms of different sizes together (see Coll-Martínez 2019; Fahmi et al. 2016; Lazzeretti et al. 2008, among others).
